# Enhanced expressions of neurodegeneration-associated factors, UPS impairment, and excess Aβ accumulation in the hippocampus of mice with persistent cerebral toxocariasis

**DOI:** 10.1186/s13071-017-2578-6

**Published:** 2017-12-22

**Authors:** Chia-Mei Chou, Yueh-Lun Lee, Chien-Wei Liao, Ying-Chieh Huang, Chia-Kwung Fan

**Affiliations:** 1 Graduate Institute of Medical Sciences, College of Medicine, Taipei Medical University, 250 Wuxing St, Taipei, 11031 Taiwan; 2 Department of Molecular Parasitology and Tropical Diseases, School of Medicine, College of Medicine, Taipei Medical University, 250 Wuxing St, Taipei, 11031 Taiwan; 3 Department of Microbiology and Immunology, School of Medicine, College of Medicine, Taipei Medical University, 250 Wuxing St, Taipei, 11031 Taiwan; 4 Research Center of International Tropical Medicine, College of Medicine, Taipei Medical University, 250 Wuxing St, Taipei, 11031 Taiwan; 5 Tropical Medicine Division, International PhD Program in Medicine, College of Medicine, Taipei Medical University, 250 Wuxing St, Taipei, 11031 Taiwan

**Keywords:** Cerebral toxocariasis, *Toxocara canis*, Zoonosis, Mice, Hippocampus, Amyloid β, Ubiquitin-proteasome system, Neurodegeneration

## Abstract

**Background:**

Toxocariasis is a worldwide zoonotic parasitic disease mainly caused by *Toxocara canis*. Humans can be infected by accidental ingestion of *T. canis* embryonated ovum-contaminated food, water, or encapsulated larvae in paratenic hosts’ viscera or meat. Since humans and mice are paratenic hosts of *T. canis*, the wandering larvae might cause mechanical tissue damage and excretory-secretory antigens may trigger inflammatory injuries to local organs. Long-term residence of *T. canis* larvae in a paratenic host’s brain may cause cerebral toxocariasis (CT) that contributes to cerebral damage, neuroinflammation and neuropsychiatric disorders in mice and clinical patients. Since the hippocampus has been long recognized as being responsible for learning and memory functions, parasitic invasion of this site may cause neuroinflammatory and neurodegenerative disorders. The present study intended to assess pathological changes, expressions of neurodegeneration-associated factors (NDAFs), including transforming growth factor (TGF)-β1, S100B, glial fibrillary acidic protein (GFAP), transglutaminase type 2 (TG2), claudin-5, substance P (SP) and interleukin (IL)-1β, and the ubiquitin-proteasome system (UPS) function in the hippocampus and associated cognitive behavior in ICR mice orally inoculated with a high, medium or low-dose of *T. canis* embryonated ova during a 20-week investigation.

**Results:**

Results indicated although there were insignificant differences in learning and memory function between the experimental mice and uninfected control mice, possibly because the site where *T. canis* larvae invaded was the surrounding area but not the hippocampus per se. Nevertheless, enhanced expressions of NDAF, persistent UPS impairment and excess amyloid β (Aβ) accumulation concomitantly emerged in the experimental mice hippocampus at 8, 16 and 20 weeks post-infection.

**Conclusions:**

We thus postulate that progressive CT may still progress to neurodegeneration due to enhanced NDAF expressions, persistent UPS impairment and excess Aβ accumulation in the hippocampus.

**Electronic supplementary material:**

The online version of this article (10.1186/s13071-017-2578-6) contains supplementary material, which is available to authorized users.

## Background

Toxocariasis, a cryptic zoonotic parasitic disease found worldwide, results from infections by roundworms*,* presumably mainly by *Toxocara canis* and to a lesser extent by *T. cati* [1, 2]. The Centers for Disease Control and Prevention also highlighted it as one of the five major indigenous neglected parasitic infections (NPIs) that should be comprehensively controlled in the USA [3]. Humans can be infected by accidental ingestion of foods, water, or soil contaminated by *T. canis* embryonated ova or larvae encapsulated in the viscera or muscles of paratenic hosts e.g. chickens or lambs [2]. The worldwide seroprevalence of toxocariasis ranges 6.3–86.8%, indicating the profound impact of *Toxocara* on global human health, and was reported to be highly related to various risk factors, including poor personal hygiene, contact with dogs or cats, and consumption of foods contaminated by eggs or encapsulated larvae of *Toxocara* [4–6]. When paratenic hosts, including mice and humans, accidentally ingest infective *T. canis* ova or tissue-encapsulated larvae, third-stage *T. canis* larvae emerge from the eggs and penetrate through the submucosa of the small intestine to further migrate to the liver via the portal circulation; they subsequently further invade various internal organs such as the liver or lungs, causing visceral larva migrans (VLM), the eyes, causing ocular larva migrans (OLM), or the central nervous system (CNS) leading to neurotoxocariasis (NT) [2, 7]. Because of improvements in diagnostic methods, human cerebral toxocariasis (CT) cases with eosinophilic meningitis, encephalomyelitis, or even seizures, recently have been commonly reported [8–10]. Several clinical studies indicated some neurotropic infections may be closely associated with various cognitive deficits, e.g. a lack of developmental progress in speech, cognitive deficits possibly indicative of dementia, and impairments of mental fluency and short-term working memory spans [11, 12]. However, *T. canis* larval invasion of the brain that causes CT rarely induces recognizable neurological signs [13]; thus, the impacts of CT on cognitive development in humans remain unanswered because very few clinical or population-based studies have examined the relationship between neuropsychological defects and CT [13]. Considering that the frequency of humans exposed to *Toxocara* is fairly high but few clinical CT cases are reported, it suggests that CT may be underestimated or ignored [1]. The number of CT cases described in the literature is still small, which might be explained by humans harboring few *Toxocara* larvae in their brains, and thus the effects of brain involvement are too cryptic to be easily explained or observed in human CT [14].

Substantial studies indicated that mice are useful animal models to explore the impacts of *T. canis* on the biology of the brain, behavioral changes and the pathogenesis of CT [13]. Our previous study indicated enhanced expressions of neurodegeneration-associated factors (NDAF) including glial fibrillary acidic protein (GFAP), transforming growth factor (TGF)-β1, S100B, transglutaminase type 2 (TG2), β-amyloid precursor protein (AβPP), neurofilament light chain (NF-L) and total (t)-tau, as well as impairments of the blood-brain barrier (BBB) and ubiquitin-proteasome system (UPS) in brains of ICR mice inoculated with a single dose of 250 eggs during an 8-week investigation [15, 16]. In addition, some studies indicated that the longevity of *T. canis* larvae resident in the brains of black chimpanzees can be up to 10 years [17], and larvae recovered from *T. canis*-infected mice brains were still alive at 465 days post-infection (dpi) [18], suggesting that the longevity of *T. canis* larvae resident in paratenic hosts is quite extended. One study also indicated that the explorative ability, response to novelty and memory function become stunted in *T. canis*-infected mice [19], indicating that these abnormal behavioral changes are likely related to the site where *T. canis* larvae invaded the brain. Since substantial studies have indicated that the cerebral hippocampal region is mainly responsible for learning and memory functions and the occurrence of neurodegenerative disorders is often accompanied by some insults like infection to this specific site [20, 21], it would be helpful to explore any abnormal behavioral changes, such as learning and memory deficits, caused by immunopathological injury to the hippocampal region.

The present study intended to assess pathological changes, NDAF expressions and UPS function in the hippocampal region as well as cognitive behavior in ICR mice orally inoculated with a high-, medium- or low-dose of *T. canis* eggs for a 20-week investigation.

## Methods

### Source of *T. canis* embryonated ova

The method for preparing embryonated *T. canis* ova was described in a previous study [22]. Briefly, adult *T. canis* worms were collected from puppies treated with antihelmintic drugs. After they were confirmed to be *T. canis* by morphological identification, the eggs were collected from the uteri of female adult worms. About a 1 cm long section of the uterus near the vulva of a female adult worm was collected and shaken in 2% formalin at room temperature to prepare a *T. canis* ova suspension. The embryonation status of *T. canis* eggs was examined every 2–3 days by microscopy to confirm whether they had successfully developed into the embryonated stage. Once the eggs had fully embryonated, they were further stored at 4 °C until use.

### Experimental procedure of animal studies

ICR mice aged 6–8 weeks were obtained from BioLASCO (Taipei, Taiwan). Mice were housed in the Laboratory Animal Center of Taipei Medical University (TMU; Taipei, Taiwan) and maintained on commercial pellet food and water ad libitum. The experimental design of *T. canis* inoculum was modified from previous studies [16, 19]. Briefly, mice were randomly divided into four groups (eight to ten mice per group), comprised of three experimental groups of mice inoculated with low- (250 *T. canis* embryonated ova), medium- (500 *T. canis* embryonated ova) and high-dose infective ova (1000 *T. canis* embryonated ova), as well as one control group of uninfected mice.

Prior to sacrifice by heart puncture at 10 dpi, and 8, 16 and 20 weeks post-infection (wpi), mice were assessed for learning and memory capacity using a Morris water maze (MWM) test. After sacrifice, three mice were used for larval recovery studies and the remaining five to seven mice per group were used for histopathological studies and assessments of NDAF expressions. The left brain of each mouse containing the hippocampal area was processed by paraffin embedding and further examined for pathological changes. The distribution of larvae in the hippocampus on three slides of each sample from the left brain was examined. The hippocampal area of the right brain was assessed for NDAF expressions, Aβ accumulation and UPS function.

### Assessment of learning and memory capacity by the MWM test

**Fig. 1:**
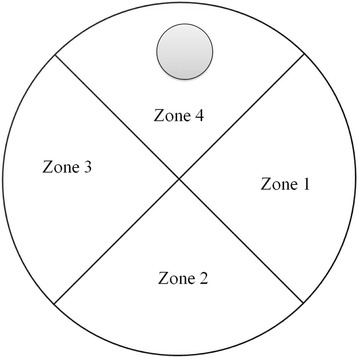
Apparatus of the Morris water maze (MWM) test and area divisions for different directions. The circle in zone 4 represents the hidden platform

To measure whether there were spatial learning and memory deficits in brains of mice infected with *T. canis*, the MWM test, a commonly used method to assess behavioral changes in learning and memory capacity in the neurosciences [23], was employed in this study. Baseline data for the learning and memory capacities of each mouse in each group were evaluated for 1 week prior to sacrifice. The MWM test training protocol included 1 day of training for platform recognition using a visible platform mode and then a continuous 4-day trial of spatial conditioning using a hidden-platform mode. Briefly, a circular pool measuring 120 cm in diameter and 50 cm deep and a round platform of 10 cm in diameter were used for the MWM test. The platform was placed inside the circular pool, which was then filled with water until the platform was 0.5 to 1 cm above the water level. The purpose of the 1-day training protocol for platform recognition with the visible platform mode was to discipline these mice to be familiar with the platform location and further that they would be able to find and climb onto the visible platform from four different directions. The cutoff time for each mouse to find and climb onto the visible platform was 60 s, and if the time was less than 60 s, the mouse was allowed to stay on the top of the visible platform; however, if a mouse could not find the visible platform within 60 s, it was placed on the top of the platform for 30 s. After completion of the 1-day visible platform training, 4 days of the subsequent hidden- platform trial for monitoring spatial conditioning commenced. During the trial, the platform was placed 0.5 to 1 cm beneath the water surface, and the cutoff time was the same used in the 1-day visible platform training program. Detailed training procedures of the 4-day hidden-platform trial are shown in Fig. 1 and Table 1. A behavioral analysis of an animal’s learning and memory capacity, e.g. the escape latency time, was performed using WaterMazeScan software (Clever Systems, Reston, VA, USA).

**Table 1:** Four-day hidden-platform protocol

	Day 1	Day 2	Day 3	Day 4
Trial 1	1	3	2	1
Trial 2	3	1	1	3
Trial 3	2	2	3	2
Trial 4	1	3	2	1

*Key*: 1, zone 1; 2, zone 2; 3, zone 3

### Larval recovery from *T. canis*-infected mouse brains

A larval recovery method described previously [16] was used to confirm whether or not *T. canis* larvae had invaded mouse brains. Briefly, brain tissue from each *T. canis*-infected mouse was cut into small pieces and individually digested in 25–30 ml of an artificial acidic pepsin/HCl solution (pH 1–2, Sigma-Aldrich, St. Louis, MO, USA) in a modified Baermann apparatus at 37 °C for at least 2 h. Subsequently, the filtered solution was poured into a Petri dish, and numbers of *T. canis* larvae were counted with an inverted microscope (Olympus, Tokyo, Japan) at 100× magnification.

### Histopathological studies of the hippocampal region

The left brain specimen of each mouse containing the hippocampal region was fixed in 10% neutral formalin for at least 12 h and embedded in paraffin; several 5 μm-thick sections were stained with hematoxylin and eosin (H&E) to assess histopathological changes.

### TGF-β1, GFAP, TG2, S100B, substance P (SP) and claudin-5 expressions as well as UPS function were assessed by western blotting (WB)

The WB procedure from a previous study [16] was applied. The hippocampal region of the right brain of each mouse was removed and immediately stored at -80 °C until use. Frozen hippocampal specimens from the same experiment group were pooled together and further homogenized and lysed in radioimmunoprecipitation assay (RIPA) buffer containing 1% of a protease inhibitor cocktail (Sigma-Aldrich, Darmstadt, Germany) at 4 °C for at least 1 h; protein supernatants were then harvested by centrifugation at 13,000× *rpm* and 4 °C for 10 min. Thereafter, the protein concentration was calculated using the Bradford method with a Bio-Rad protein assay kit (Life Sciences, Taipei, Taiwan). Subsequently, 50 μg of each protein sample was boiled for 5 min to denature the protein, then further separated by 12% sodium dodecylsulfate polyacrylamide gel electrophoresis (SDS-PAGE) and finally transferred onto a 0.45 μm pore size polyvinylidene fluoride (PVDF) membrane for 2 h. Membranes were blocked with 10% skim milk in Tris-buffered saline with Tween 20 (TBST) for 4 °C overnight. Primary antibodies, including a mouse anti-β-actin monoclonal antibody (mAb) (1:10,000, cat. no. A2228, Sigma-Aldrich), mouse anti-TGF-β1 mAb (1:200, cat. no. T0438, Sigma-Aldrich), mouse anti-SP mAb (1:200, cat. no. ab14184, Abcam, Cambridge, MA, USA), mouse anti-GFAP mAb (1:200, cat. no. G3893, Sigma-Aldrich), goat anti-TG2 polyclonal antibody (pAb) (1:1000, cat. no. T7066, Sigma-Aldrich), mouse anti-S100B mAb (1:200, cat. no. S2532, Sigma-Aldrich), rabbit anti-claudin-5 pAb (1:100, cat. no. sc-28,670, Santa Cruz Biotechnology, Dallas, TX, USA), mouse anti- interleukin (IL)-1β (3A6) mAb (1:1000, cat. no. 12242, Cell Signaling Technology, Danvers, MA, USA) and mouse anti-ubiquitin mAb (1:100, cat. no. Mab1510, Chemicon, Billerica, MA, USA), were added at 37 °C for hybridization for 2 h. After washing with TBST several times, membranes were further incubated with secondary antibodies of horseradish peroxidase (HRP)-conjugated immunoglobulin G (IgG), including rabbit anti-mouse IgG (cat. no. A9044, Sigma-Aldrich), goat anti-rabbit IgG (cat. no. A0545, Sigma-Aldrich), or donkey anti-goat IgG (cat. no. sc-2020, Santa Cruz Biotechnology), at 1:10,000 dilutions. Immunoreactions were detected with a Western Lightning ECL Pro kit (PerkinElmer, Waltham, MA, USA), and thereafter the densities of immunoreactive bands were measured using a UVP Biospectrum AC System (UVP, Upland, CA, USA) in the Core Facility Center of TMU. Reactive bands of TGF-β1, SP, GFAP, TG2, S100B, claudin-5, IL-1β precursor, IL-1β and β-actin, were detected at 25, 47, 51, 78, 21, 24, 31, 17 and 42 kDa, respectively.

### Aβ aggregation detection by modified WB via semi-denaturing detergent-agarose gel electrophoresis (SDD-AGE)

The SDD-AGE method for amyloid aggregation detection was described previously [24]. Briefly, 50 μg of total proteins from each sample was boiled and centrifuged as previously described, then loaded onto a 1.5% agarose gel with a TBE buffer running system. A protein ladder was used to check the gel running condition. The Aβ protein fragment 1-40 (cat. no. A1075, Sigma-Aldrich) was loaded as a positive control in the hybridization step. Proteins were transferred to PVDF membranes employing a capillarity method at room temperature overnight. Subsequent procedures were the same as those used in routine WB steps, and the added primary antibody was a mouse anti-Aβ mAb at a 1:1000 dilution (cat. no. A5213, Sigma-Aldrich).

### Statistical analysis

All data are presented as the mean value with the standard deviation (SD). The statistical difference in escape latency of the MWM test between the experimental and uninfected groups was assessed by a two-way analysis of variance (ANOVA) with a Bonferroni *post-hoc* test as calculated with GraphPad Prism 5 software (GraphPad Software, La Jolla, CA, USA); while statistical differences in NDAF expressions, Aβ accumulation and UPS function with either different infection doses or infection times were evaluated by a one-way ANOVA with *post-hoc* analysis performed by using Tukey’s multiple comparison tests. All data in different infection time in mice with low-, medium- and high-dose infections were compared with a control which was the average value of uninfected groups at 10 dpi and 20 wpi. A *P*-value of < 0.05 was considered a significant difference.

## Results

### Insignificantly longer escape latency times in experimental mice with low-, medium- and high-dose infections than that in uninfected control mice

**Fig. 2:**
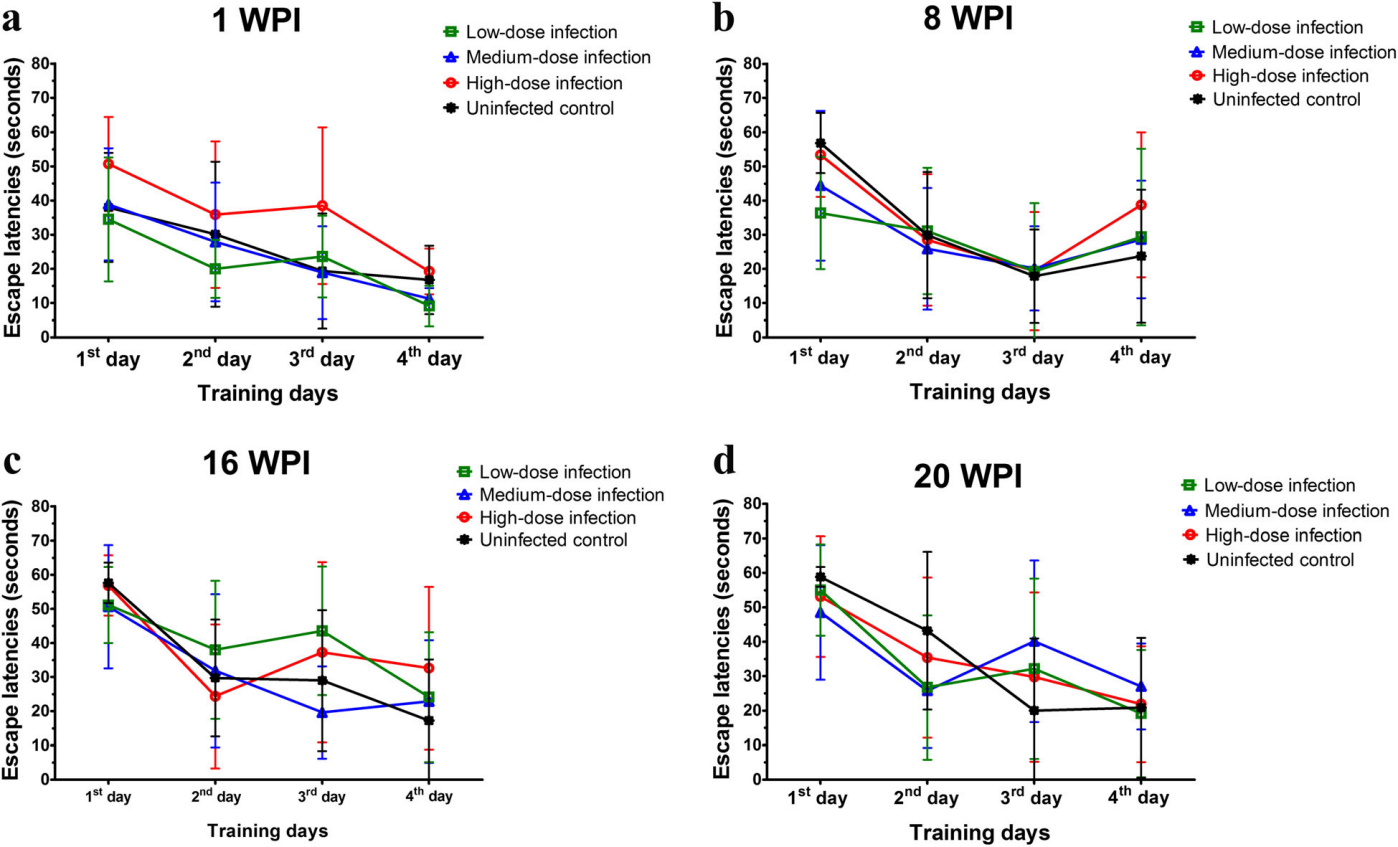
Insignificantly different escape latencies in mice with low-, medium- and high-dose infections compared to those in uninfected control mice at 1 (**a**), 8 (**b**), 16 (**c**) and 20 (**d**) weeks post-infection (wpi)

The escape latency is an index which is widely used in the MMW test to reveal spatial learning and memory changes in animal behavioral studies. Escape latencies of mice at 1, 8, 16 and 20 wpi in this study are shown in Fig. 2. There was a longer escaped latency performance by mice with high inoculum of *T. canis* ova on the second and third training days compared to the other groups at 1 wpi (Fig. 2a) but it was not statistically significant (*F*_(9,112)_ = 0.49, *P* = 0.8773). Although longer escape latencies were also found in experimental groups with low-, medium- and high-dose infections than those in uninfected control mice at 8, 16 and 20 wpi, they did not statistically significantly differ at 8 (*F*_(9,112)_ = 0.84, *P* = 0.5847), 16 (*F*_(9,108)_ = 1.04, *P* = 0.4125), or 20 wpi (*F*_(9,100)_ = 1.00, *P* = 0.4465) (Fig. 2b-d). Overall, these data imply that there were not significant behavioral defects in learning and memory function in mice brain with persistent *T. canis* larval infection.

### *Toxocara canis* larvae were found in brain of mice infected with low-, medium- and high-doses at 8, 16 and 20 wpi

**Fig. 3:**
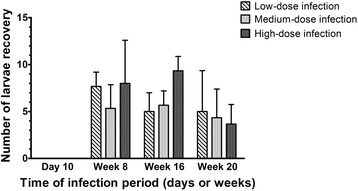
*Toxocara canis* larvae recovered from mouse brains with low-, medium- and high-dose infections at 10 days post-infection (dpi) and 8, 16 and 20 weeks post-infection (wpi)

Results of larval recovery from the brain are shown in Fig. 3. No larvae were found at 10 dpi. Average numbers (mean ± SD) of *T. canis* larvae recovered at 8 wpi were 7.7 ± 1.5, 5.3 ± 2.5 and 8.0 ± 4.6; at 16 wpi numbers were 5.0 ± 2.0, 5.7 ± 1.5 and 9.3 ± 1.5; and at 20 wpi, recovered larval numbers were 5.0 ± 4.4, 4.3 ± 3.1 and 3.7 ± 2.1 in low-, medium- and high-dose infected mice, respectively.

### *Toxocara canis* larvae were found in the vicinity of the hippocampal region of mice brains in the experimental groups

**Fig. 4:**
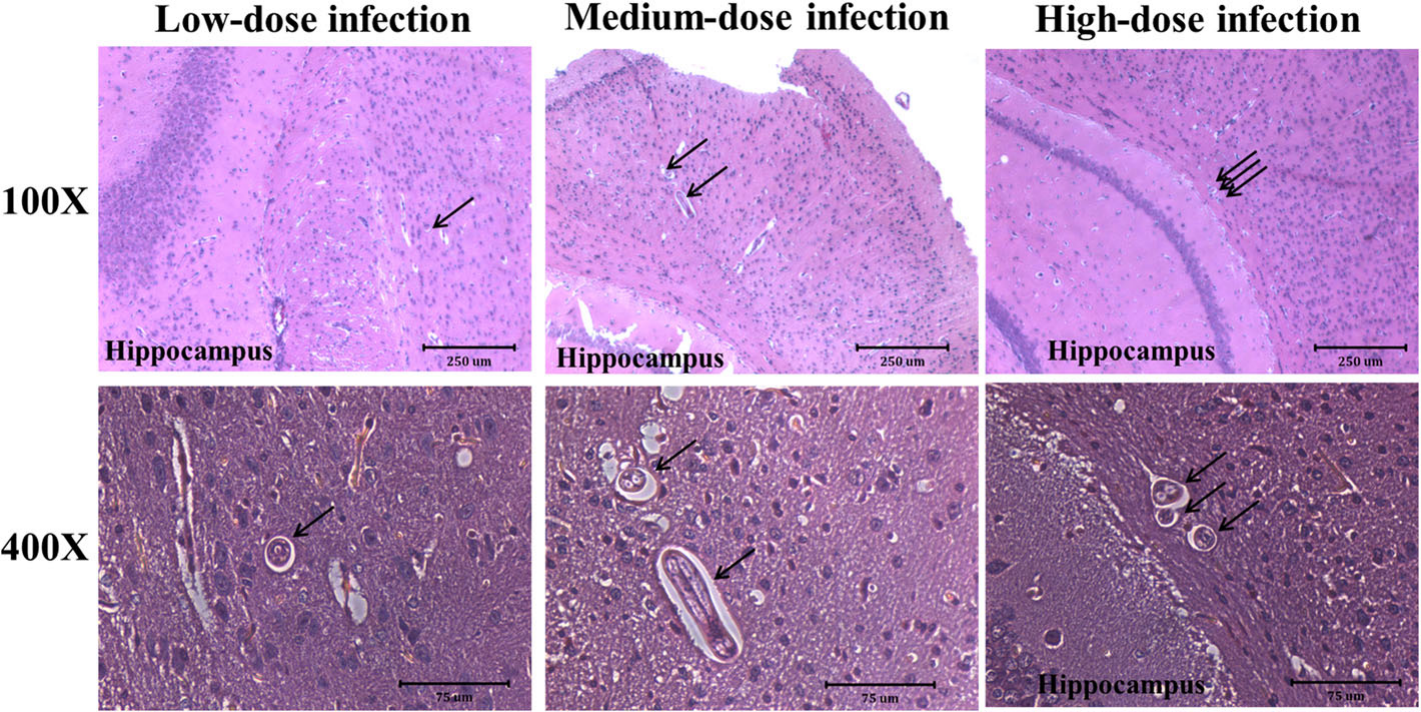
H&E-stained section of a mice hippocampus from infected mice with low-, medium- and high-dose infections. *Toxocara canis* larval sections (*arrow*) were found near the hippocampus at 16 weeks post-infection (wpi). No *T. canis* larvae were found inside hippocampal sections in mice with low-, medium- and high-dose infections. *Scale-bars*: 100× magnification, 250 μm; 400× magnification, 75 μm

Histopathological findings from brain sections of *T. canis*-infected mice are shown in Fig. 4. *Toxocara canis* larvae were present in areas near the hippocampus at 16 wpi (Fig. 4). No *T. canis* larvae were found to have invaded the hippocampal region of any brain sections in mice with low-, medium- or high-dose infection at 10 dpi, or 8, 16 or 20 wpi (data not shown). However, the inflammatory infiltrate and tissue damage in the hippocampus and other brain regions were not found in the experimental groups.

### Enhanced NDAF expressions in hippocampal areas of experimental groups of mice

**Fig. 5:**
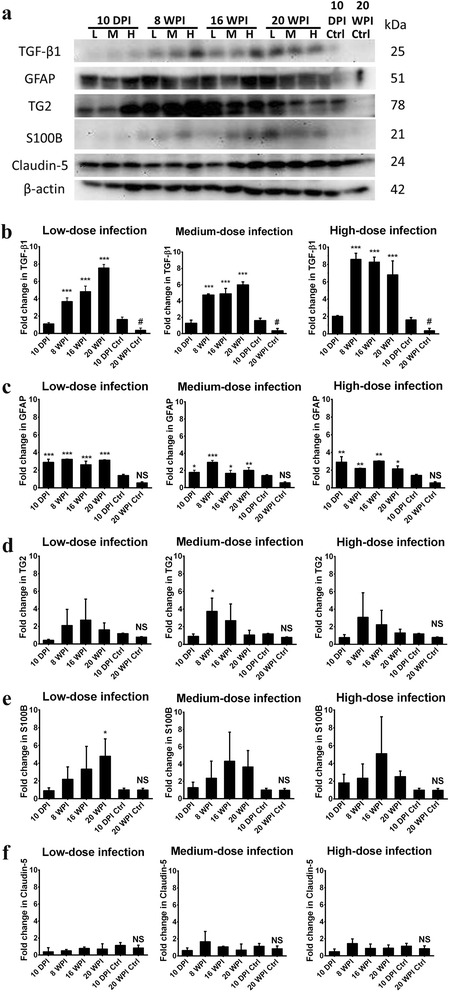
Neurodegeneration-associated factor (NDAF) expressions, including transforming growth factor (TGF)-β1, glial fibrillary acidic protein (GFAP), transglutaminase type 2 (TG2), S100B and claudin-5, increased in the hippocampus of *Toxocara canis*-infected mice inoculated with low-, medium- and high-dose infections at 8, 16 and 20 weeks post-infection (wpi). **a** Protein expression levels of NDAFs assessed by Western blotting. **b-f** Quantification of NDAF protein expressions represented as the mean with SD. *P < 0.05, **P < 0.01 and ***P < 0.001 indicate a significant difference with uninfected control mice at 10 days post-infection (dpi) and 20 wpi. ^#^Indicates a significant difference (*P* < 0.05) between 10 dpi- and 20 wpi-uninfected control mice. *Abbreviation*: NS, no significant difference between 10 dpi- and 20 wpi-uninfected control mice

**Fig. 6:**
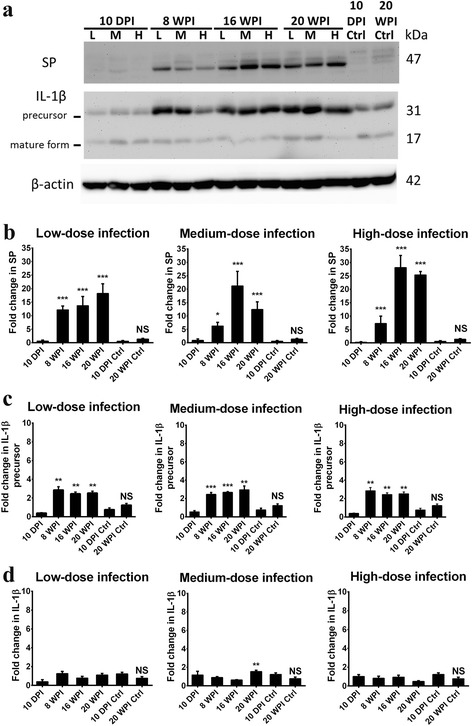
Levels of substance P (SP), the interleukin (IL)-1β precursor and IL-1β, which were also correlated with the neuroinflammatory response, were higher in the hippocampus of *Toxocara canis*-infected mice inoculated with low-, medium- and high-dose infections at 8, 16 and 20 weeks post-infection (wpi). **a** Protein accumulation in the mouse hippocampus was analyzed by Western blotting. **b-d** Quantification of SP, the IL-1β precursor, and IL-1β accumulation in the hippocampal region. Data are presented as the mean with SD. *P < 0.05, **P < 0.01 and ***P < 0.001 indicate a significant difference with uninfected control mice at 10 days post-infection (dpi) and 20 wpi. *Abbreviation*: NS, no significant difference between 10 dpi- and 20 wpi-uninfected control mice

NDAF expressions related to cerebral damage, neuronal inflammation and degeneration in mice hippocampal areas invaded by *T. canis* larvae in the experimental and uninfected control groups of mice are shown in Figs. 5 and 6. Except at 10 dpi, TGF-β1 expression had significantly increased at 8, 16 and 20 wpi in all experimental groups of mice. Quantitatively, it had sharply increased by 3.68-, 4.83- and 7.56-fold in mice with low-dose infection, by 4.76-, 4.90- and 6.00-fold at 8, 16 and 20 wpi in mice with medium-dose infection, and by 8.60-, 8.28- and 6.81-fold at 8, 16 and 20 wpi in mice with high-dose infection at 8, 16 and 20 wpi, respectively. It seemed likely that there was a time-dependent infection effect on TGF-β1 expression in mice with low-dose infection. Surprisingly, an increase of > 15-fold was found in mice with all doses of infection compared to that in uninfected mice group at 20 wpi (Additional file 1: Figure S1a). These data suggest that *T. canis* larval invasion could profoundly trigger TGF-β1 expression in the hippocampus in the late infection stage (Fig. 5b).

Levels of GFAP in the hippocampal area began to slightly increase, reaching nearly 2.91-, 3.24-, 2.64- and 3.15-fold higher at 10 dpi and 8, 16 and 20 wpi, respectively, in mice with low-dose infection. Comparable results were shown at 10 dpi and 8, 16 and 20 wpi in mice with medium- and high-dose infections (Fig. 5c). In mice with different infection doses at 20 wpi, the highest GFAP protein level with a 5.43-fold increase was expressed in the hippocampus of mice with low-dose infection, while 3.49- and 3.71-fold increases were shown in in mice with medium- and high-dose infections, respectively (Additional file 1: Figure S1b).

Even though TG2 expressions had slightly increased by 2.12- and 3.08-fold at 8 wpi in mice with low- and high-dose infections, respectively, a significant increase was only found a 3.76-fold at 8 wpi in mice with a medium-dose infection (Fig. 5d). S100B was sharply expressed in the late infection stage in mice with low-, medium- and high-dose infections; however a significant 4.80-fold increase was only evident in mice with low-dose infection at 20 wpi (Fig. 5e).

The tachykinin neuropeptide, SP, significantly increased from 8 wpi and remained high until 16 and 20 wpi in mice with low-, medium- and high-dose infections. There were drastic 12.18-, 13.71- and 18.21-fold increases in the low-dose infection group, 6.26-, 21.23- and 12.43-fold increases in the medium-dose infection group, a7.26-, 28.12- and 25.36-fold increases in mice with high-dose infection at 8, 16 and 20 wpi, respectively (Fig. 6b). In mice with different infection doses at 20 wpi, SP exhibited 13.02-, 8.89- and 18.12-fold increases in the low-, medium- and high-dose infection groups, respectively (Additional file 2: Figure S2a).

IL-1β is initially synthesized as the precursor protein with a molecular weight of 31 kDa, and after cleavage, it becomes the 17-kDa bioactive form. In Fig. 6, levels of the IL-1β precursor had significantly increased, by 2.84-, 2.44- and 2.52-fold in mice with low-dose infection, by 2.46-, 2.68- and 2.95-fold in mice with medium-dose infection and by 2.85-, 2.44- and 2.52-fold in high-dose infection group at 8, 16 and 20 wpi, respectively (Fig. 6c). On the other hand, there were 2.04-, 2.38- and 2.84-fold changes in IL-1β precursor protein expressions in mice with low-, medium- and high-dose infections at 20 wpi, respectively (Additional file 2: Figure S2b). However, expression of the active form of IL-1β was evident with a 2.00-fold increase at 20 wpi only in mice with a medium-dose infection (Fig. 6d).

On the contrary, different from other NDAFs with high expression in the early or late infection period, the level of major components of tight junction proteins of the BBB, claudin-5, had slightly increased in mice with medium- and high-dose infections, reaching 1.68- and 1.46-fold at 8 wpi (*P* > 0.05), respectively (Fig. 5f). Altogether, these data suggest that *T. canis* larval invasion of the hippocampus can profoundly enhance most NDAF expression profoundly in the late infection stage e.g. at 20 wpi, irrespective of the infective inoculum level.

### Persistent UPS impairment and enhanced Aβ accumulation in the hippocampus of infected mice at 8, 16 and 20 wpi

**Fig. 7:**
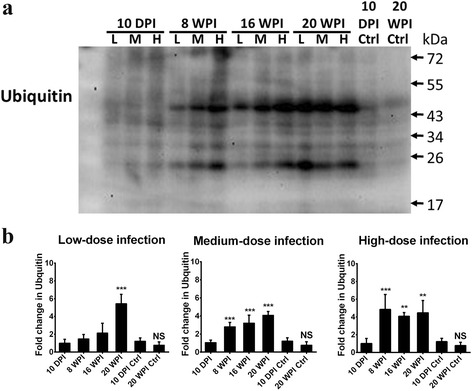
Impairment of the ubiquitin-proteasome system (UPS) was enhanced in the hippocampus of *Toxocara canis*-infected mice inoculated with low-, medium- and high-dose infections at 8, 16 and 20 weeks post-infection (wpi). **a** Ubiquitin accumulation in the mice hippocampus as analyzed by Western blotting. **b** Quantification of ubiquitin accumulation in hippocampal regions of mice given low-, medium- and high-dose infections. Data are presented as the mean with SD. *P < 0.05, **P < 0.01 and ***P < 0.001 indicate a significant difference with uninfected control mice at 10 days post-infection (dpi) and 20 wpi. *Abbreviation*: NS: no significant difference between 10 dpi- and 20 wpi-uninfected control mice

Persistent aberrant expressions of ubiquitin and ubiquitylated proteins in the hippocampal area are shown in Fig. 7. Compared to ubiquitin levels in uninfected control mice, those in the hippocampus of ubiquitin levels in low-dose infected mice showed a 5.44-fold increase at 20 wpi. With a medium-dose infection, levels had obviously increased by 2.81-, 3.22- and 4.08-fold at 8, 16 and 20 wpi, respectively, while with a high-dose infection, levels reached 4.85-, 4.08- and 4.48-fold at 8, 16 and 20 wpi, respectively (Fig. 7b). Comparing of different infection doses at 20 wpi, ubiquitin levels rose sharply in mice with low-, medium- and high-dose infections at 20 wpi, reaching 6.99-, 5.10- and 5.75-fold increases, respectively (Additional file 3: Figure S3).

**Fig. 8:**
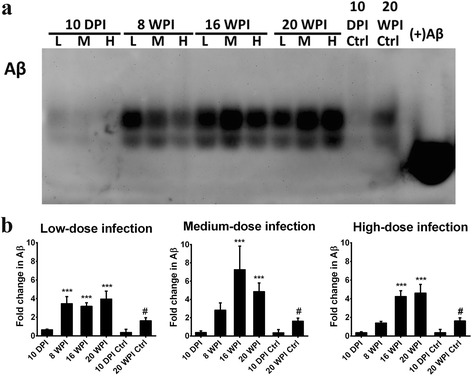
β-Amyloid (Aβ) accumulated in the hippocampus of *Toxocara canis*-infected mice at 16 and 20 weeks post-infection (wpi). **a** Aβ accumulation in the *T. canis*-infected mice hippocampus was assessed by agarose gel-modified Western blotting. **b** Quantification of Aβ accumulation in the hippocampal region of mice with low-, medium- and high-dose infections, presented as the mean with SD. *P < 0.05, **P < 0.01 and ***P < 0.001 indicate a significant difference with uninfected control mice. ^#^Indicates a significant difference (*P* < 0.05) between 10-days post-infection (dpi) and 20-wpi uninfected control mice

Expression levels of insoluble Aβ in the mice hippocampus are shown in Fig. 8. In mice with low-dose infection, insoluble Aβ expression was significantly enhanced in the mouse hippocampus at 8, 16 and 20 wpi with increases of 3.48-, 3.19- and 3.97-fold, respectively. In the medium- and high-dose groups, expressions of insoluble Aβ were significantly enhanced at 16 and 20 wpi, and were 7.27- and 4.87-fold higher in mice with medium-dose infection and were 4.25- and 4.63-fold in mice with high-dose infection, respectively (Fig. 8b). Accumulation of insoluble Aβ was significantly higher in mice at 20 wpi, at 2.43-, 2.99- and 2.84-fold increases in mice with low-, medium- and high-dose infections, respectively (Additional file 4: Figure S4).

Our results suggest that UPS impairment and insoluble Aβ aggregations occurred in the hippocampus of mice with prolonged *T. canis* infection, irrespective of the infective inoculum level.

## Discussion

Despite the clinical and pathological features of human CT having been described [8–10, 14, 25], connections of CT with neuroinflammation and cognitive impairment remain largely unclear. Alzheimer’s disease (AD), an age-related neurodegenerative disorder, is the most common dementia in the world that occurs mainly in elderly people [26]. Extracellular amyloid plaques composed of neurotoxic Aβ aggregations, intracellular neurofibrillary tangles (NFTs) assembled by hyperphosphorylated tau protein, and UPS dysfunction unable to clear toxic or unwanted proteins that can cause neuroinflammation and eventually neuronal death, have all been hypothesized to play major roles in the pathogenesis of AD [26].

Several cytokines, glial proteins, and enzymes with elevated expressions, such as TGF-β1, S100B, GFAP and TG2, are highly suspected of being involved in traumatic brain injury (TBI), neuroinflammation and acute CT [16], and also in the pathogenesis of AD [27]. GFAP, an intermediate filament protein particularly expressed by astrocytes in the CNS, plays an important role in astrocyte-neural interactions and is highly responsible for astrogliosis after CNS injuries and neurodegeneration [20]. Since some studies indicated that S100B manipulates neurite outgrowth, neuronal inflammation and apoptosis, overexpression of S100B is highly associated with activation of astrogliosis and also neuronal loss in AD [21], while such effects can be attenuated by blocking S100B. Studies also indicated that TG2, a calcium-dependent enzyme, contributes to wound healing, inflammation, and physical cross-linking in protein-protein interactions, and additionally, it is involved in some neurodegenerative diseases including AD [28]. Increased TG2 was implied to enhance NFT formation and promote low-soluble complex formation, aggregations of α-synuclein, and Aβ peptide cross-linking [29]. TGF-β1 is physiologically expressed in the hippocampus and other brain regions involved in brain development and modulation of synaptic transmission [30]. Increased TGF-β1 expression reportedly functions as a potential neuroprotective cytokine as its anti-inflammatory, antiapoptotic and neuronal-regenerative effects attenuate brain ischemia, TBI and neurodegenerative disorders [30, 31]; substantial evidence also revealed that a high TGF-β1 expression is a pathogenic factor in AD development and progression. An investigation suggested that long-term overexpression of TGF-β1 may cause hippocampal structural transformation and learning deficits [20]. The other study also indicated that TGF-β1 may accelerate the risk of late-onset AD and AD-related depression [32], and in addition it was highly implicated in causing Aβ plaque formation and cognitive and cerebrovascular alterations [33, 34]. Another group demonstrated that blockage of TGF-β1 downstream signaling could attenuate AD-like pathology [35]. The present study found that TGF-β1, TG2, S100B and GFAP were significantly elevated during early infection at 8 wpi (Fig. 5), which was similar to results of our previous study [16]. Meanwhile, we also found significant increases in GFAP in the mice hippocampus, beginning early at 10 dpi, remaining stable and high until 20 wpi, irrespective of the inoculum level or infection time (Fig. 5). This implies that astrogliosis might emerge from early infections beginning as early as 10 dpi and continue to late infection at 20 wpi of the experiment. Interestingly, expressions of S100B and TGF-β1 in the mouse hippocampus were concomitantly enhanced in mice with medium and high infections (Fig. 5), although no significant difference in larval numbers was found in brains of mice between different ovum inoculum groups (Fig. 3).

Despite SP, a neuropeptide of the tachykinin family, being involving in protecting cerebellar granule cells from apoptosis induced by toxic Aβ [36], substantial studies indicated that increased expressions of SP and its receptor, neurokinin-1 receptor (NK-1R), are pathologically involved in different types of neurological injuries, e.g. TBI [37], increased vascular permeability of the BBB and acute neurogenic inflammation [38], and various diseases, e.g. cancer, murine schistosomiasis [39], cysticercosis and trypanosomiasis [40]. Our study revealed that enhanced SP expression was found in the early (8 wpi) and also late stages (16 and 20 wpi) of infection (Fig. 6). Taken together, these data suggest that both TGF-β1 and SP are important pathological factors causing neurological inflammation or even neurodegeneration in chronic CT. Substantial evidence indicates that SP can be induced in T cells and macrophages and that SP-induced NK-1R internalization is regulated by TGF-β1 [39, 41]. In addition, high production of proinflammatory cytokines induced by SP can be expressed through the synergic collaboration of TGF-β1 and SP [41]. Taking these findings together, it seems likely that elevated SP expression is highly manipulated by TGF-β1. Nevertheless, clarifying the contributory roles of TGF-β1 and SP in the immunopathogenesis of CT requires more evidence, particularly of the immunopathological effects on astrocytes and microglial cells, which are responsible for neurological inflammation after brain injuries [42] and neuroinflammation [43].

The hippocampus is an important and indispensable brain region in the CNS, responsible for learning, spatial and remote memory networks, navigation and neurotransmission in interactive signal integration between the cortex and hippocampus [44], and it was revealed to be correlated with AD [45] and epilepsy [46]. Astrocytes [45] and microglial cells [42, 43] are major neuroglial cells responsible for CNS immune responses. The relationship between microglial polarization and IL-1β secretion in neuroinflammation after a TBI [42], in AD [31, 43], and in psychiatric disorders, such as major depressive disorder, bipolar disorder, autism and schizophrenia [47], has been well described. On the other hand, it was shown that the hippocampus is the major site expressing IL-1β, the IL-1β receptor (IL-1βR), and the naturally occurring IL-1βR antagonist (IL-1RA) in the brain [48]; it was further indicated that enhanced hippocampal IL-1β expression may play a pivotal role in influencing hippocampal synaptic plasticity modulation and memory impairment. High expression of IL-1β is involved in initial priming that further leads to sensitization of microglia and aging progression [31, 48]. In the present study, we found that expression of the IL-1β precursor protein was consistently elevated, starting from 8 wpi in all experimental groups of mice with an infected hippocampus; in contrast, significantly elevated expression of mature IL-1β protein was only shown at 20 wpi in mice with medium-dose infection (Fig. 6). Nevertheless, it remains largely unclear how IL-1β and other related cytokines and chemokines are involved in the immunopathogenesis of CT; thus, more experiments are needed to provide further insights into this infectious disease.

Immune-mediated neurodegeneration was recently emphasized in the pathogenesis of neurocognitive disorders and AD. However, investigations often focused on viral and bacterial infections [49], but rarely has the focus been on parasitic invasions of the brain. It was indicated that the mouse strain, *T. canis* infective dose, infection duration and different methods of assessing neurological immunopathology are important conditions for CT investigations [19]. In addition to this investigation, some investigators suggested that aberrantly increased levels of neurotransmitters and nerve fiber demyelination were highly involved in experimental CT [50]; however, behavioral changes should be examined to ascertain such causality in experimental animals with CT. One study on *T. canis*-infected inbred BALB/c mice (a strain susceptible to *T. canis* infection) inoculated with an extremely high dose of 2000 ova showed a significantly longer latency using a water-finding apparatus at 6 wpi, compared to uninfected BALB/c mice and *T. canis*-infected NIH mice (a strain resistant to *T. canis* infection) [19]. Another study also found that C57BL/6 inbred mice with CT caused by oral inoculation with 2000 *T. canis* or *T. cati* embryonated ova exhibited abnormal general activity, sensorimotor function and memory function, as assessed by a classic maze to find a food reward [51]. These finding are similar to those of neurocysticercosis (NCC), which is caused by *T. solium* larval invasion of the brain [52]. It was found that inbred BALB/c AnN (H2-d) mice with chronic cysticercosis can change their mood and behavior as assessed by an object recognition task, and levels of neurotransmitters decreased in the mouse hippocampus [52]. More importantly, several studies indicated that inoculum with *T. canis* embryonated eggs also plays an important role in affecting the *T. canis* larval burden and inflammation severity in murine toxocariasis. One study suggested that it is ideal to give an inocula of a low dose of 100 *T. canis* eggs to mice [53], and a low burden of six *T. canis* larvae present in the brain and abnormal behavior changes, e.g. learning and memory deficits in mice, is likely to more realistically reflect the situation in humans and wild rodents with CT [54].

In this study, although longer escape latencies on the third or fourth training days in outbred ICR mice with low -, medium- and high-dose inocula were found in the early stage of infection at 1 and 8 wpi and the late stage at 16 and 20 wpi, they were not statistically significant as compared to the uninfected control group of mice (Fig. 2). On the other hand, we didn’t find any inflammatory infiltrate in hippocampus and other brain region in our ICR mice model, similar to a previous study [16]. This may be because the different inbred and outbred mice models used in CT study. Nevertheless, our present study found that the enhanced NDAF expression, UPS impairment and insoluble Aβ accumulation that concomitantly occurred in the hippocampus of mice with CT strongly indicated a correlation with prolonged *T. canis* infection, irrespective of the infective inoculum level (Figs. 5, 6, 7 and 8). This means that the infection time of *T. canis* larvae invading the brain, e.g. early infection vs late infection, is more critical in influencing the severity of neurogenic inflammation and brain injury in *T. canis*-infected ICR mice, rather than the dose of *T. canis* ova. The hippocampal tissues from the left brain of mice were used for histopathological study and which from the right brain of mice were for NDAFs assessment; however, the event of *T. canis* larval migration in the left or right brain of mice is a random issue. There is still lack of evidence to reveal the different probability of larval migration between right and left brain in the paratenic hosts.

It should be noted that we also found *T. canis* larval invasion of areas near the hippocampus in mice with low-, medium- and high-dose infections at 16 wpi (Fig. 4). This finding might explain why there were no significant differences in spatial learning behaviors in the present ICR mice model, because the hippocampal area, which is responsible for learning and memory function, did not directly suffer from physical injury due to *T. canis* larval invasion. Another issue is that spatial learning behavior, which is strictly manipulated by the hippocampus, is an important indicator of the AD syndrome; however, episodic memory loss is suggested to be more sensitive in reflecting the real status of the sporadic form of AD and other non-AD dementia types in clinical practice [55]. Nevertheless, UPS impairment, enhanced NDAF expressions and insoluble Aβ accumulation were notably demonstrated in the mice hippocampus at 16 and 20 wpi (Figs. 7 and 8), suggesting a dysfunctional UPS was unable to clean unwanted or toxic proteins and insoluble Aβ, thus resulting in Aβ accumulation in the hippocampus in the long run.

## Conclusions

In conclusion, even though spatial learning behaviors were not found to be abnormal in the present study, accelerating NDAF expressions, UPS impairment and continual insoluble Aβ accumulation in the mice hippocampus imply that chronic CT may still silently progress to AD if the hippocampus is continually insulted over the long-term by *T. canis* larval chemical materials. Our study provides a novel suggestion of disease mechanisms and a correlation between chronic CT and AD progression.

## Additional files

Additional file 1: Figure S1. Increased neurodegeneration-associated factor (NDAF) expressions in the hippocampus of *Toxocara canis*-infected mice at 10 days post-infection (dpi) and 20 weeks post-infection (wpi). Data are presented as the mean with SD. *P < 0.05, **P < 0.01 and ***P < 0.001 indicate a significant difference with uninfected control mice. (TIFF 2795 kb)

Additional file 2: Figure S2. Accelerated substance P (SP), interleukin (IL)-1β precursor, and IL-1β expressions in the mouse hippocampus at 20 weeks post-infection (wpi). Data are presented as the mean with SD. *P < 0.05, **P < 0.01 and ***P < 0.001 indicate a significant difference with uninfected control mice. (TIFF 1696 kb)

Additional file 3: Figure S3. The ubiquitin-proteasome system (UPS) is impaired in experimental mouse hippocampus at 20 weeks post-infection (wpi). Data are presented as the mean with SD. **P < 0.01 and ***P < 0.001 indicate a significant difference with uninfected control mice. (TIFF 585 kb)

Additional file 4: Figure S4. β-Amyloid (Aβ) aggregation is enhanced in experimental mouse hippocampus at 20 weeks post-infection (wpi). Data are presented as the mean with SD. **P < 0.01 and ***P < 0.001 indicate a significant difference with uninfected control mice. (TIFF 577 kb)

## Abbreviations

Aβ: Amyloid β
BBB: Blood-brain barrier
CNS: Central nervous system
CT: Cerebral toxocariasis
dpi: Days post-infection
ELISA: Enzyme-linked immunosorbent assay
GFAP: Glial fibrillary acidic protein
H&E: Hematoxylin and eosin
IL: Interleukin
MWM: Morris water maze
NDAF: Neurodegeneration-associated factor
NPI: Neglected parasitic infection
NT: Neurotoxocariasis
OLM: Ocular larva migrans
SP: Substance P
TG2: Transglutaminase type 2
TGF: Transforming growth factor
UPS: Ubiquitin-proteasome system
VLM: Visceral larva migrans
WB: Western blotting
wpi: Weeks post-infection
